# Caf1 regulates the histone methyltransferase activity of Ash1 by sensing unmodified histone H3

**DOI:** 10.1186/s13072-023-00487-6

**Published:** 2023-04-29

**Authors:** Eojin Yoon, Ji-Joon Song

**Affiliations:** Department of Biological Sciences, KI for BioCentury, Korea Advanced Institute of Science and Technology (KAIST), Daejeon, 34141 Korea

## Abstract

Histone modifications are one of the many key mechanisms that regulate gene expression. Ash1 is a histone H3K36 methyltransferase and is involved in gene activation. Ash1 forms a large complex with Mrg15 and Caf1/p55/Nurf55/RbAp48 (AMC complex). The Ash1 subunit alone exhibits very low activity due to the autoinhibition, and the binding of Mrg15 releases the autoinhibition. Caf1 is a scaffolding protein commonly found in several chromatin modifying complexes and has two histone binding pockets: one for H3 and the other for H4. Caf1 has the ability to sense unmodified histone H3K4 residues using the H3 binding pocket. However, the role of Caf1 in the AMC complex has not been investigated. Here, we dissected the interaction among the AMC complex subunits, revealing that Caf1 uses the histone H4 binding pocket to interact with Ash1 near the histone binding module cluster. Furthermore, we showed that H3K4 methylation inhibits AMC HMTase activity via Caf1 sensing unmodified histone H3K4 to regulate the activity in an internucleosomal manner, suggesting that crosstalk between H3K4 and H3K36 methylation. Our work revealed a delicate mechanism by which the AMC histone H3K36 methyltransferase complex is regulated.

**Supplementary Information:**

The online version contains supplementary material available at 10.1186/s13072-023-00487-6.

## Introduction

DNA in eukaryotic cells forms into a high-order structure chromatin composed of a repeating nucleosome unit. Chromatin structures are modified in various ways to precisely control gene expression and many protein complexes are involved in chromatin modifications. The N- or C-terminal tails of histones in nucleosomes are major targets of diverse covalent modifications including methylation, acetylation, phosphorylation, ubiquitylation, and others [32]. These modifications either directly control chromatin structure or serve as a platform to recruit other transcriptional regulators [16]. Covalent histone modifications are dynamically regulated by writers and erasers and are recognized by readers via specific histone binding modules, such as the Plant homeodomain (PHD), Bromodomain (BRD), Chromodomain (CD), Bromo-adjacent homeodomain (BAH) and WD40 repeat domains [34]. Furthermore, crosstalk occurs between the histone modifications. For example, ubiquitylation of histone H2A stimulates PRC2 histone H3K27 methyltransferase activity, and histone H3K4 methylation inhibits PRC2 activity [37].

Among these modifications, histone methylation plays critical roles in activating or repressing gene expression. While H3K9 and H3K27 methylation is directly involved in gene repression, H3K4 and H3K36 methylation is involved in gene activation [3]. Ash1 in flies and ASH1L in humans are histone methyltransferases (HMTases) and belong to the Trithorax Group of proteins. The histone target site of Ash1 has been controversial. However, it has been clearly demonstrated that Ash1 is a bona-fide histone H3K36 methyltransferase [2]. The human homolog ASH1L has been linked to a number of diseases, such as Tourette’s syndrome, autism spectrum disorder, and intellectual disability [6, 21, 24, 25, 28, 42].

**Fig. 1:**
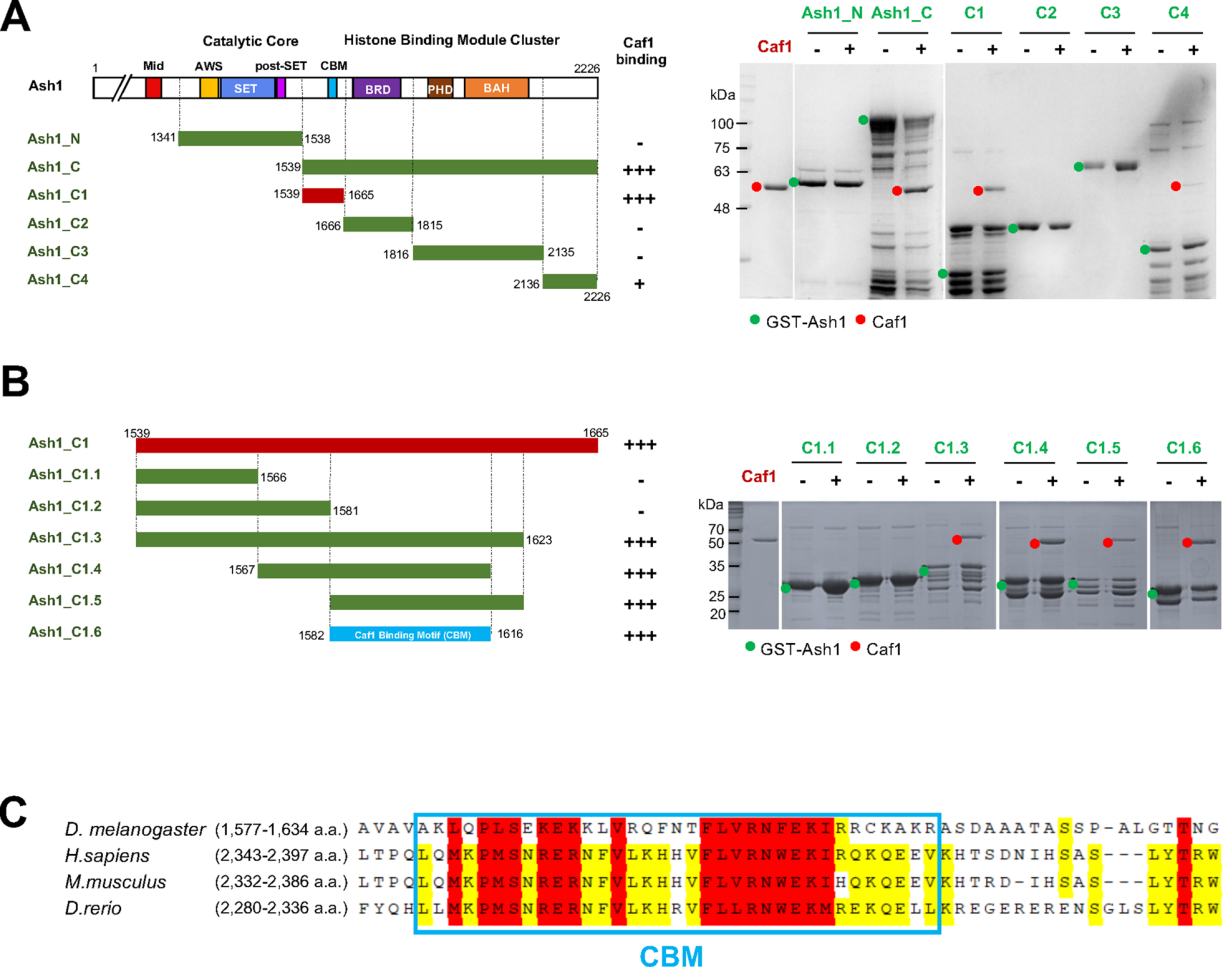
Caf1 binds to the proximal region of the Ash1 histone binding module cluster. **A** A series of the truncation constructs of Ash1 and their binding to Caf1. Strong binding is indicated with ‘+++’, weak binding with ‘+’ and no binding with ‘−’ (left panel) based on GST-pulldown experiments with GST-Ash1 constructs and Caf1 (right panel). GST-Ash1 constructs are marked in green dots and Caf1 with red dots. **B** Further truncation of Ash1_C1 and their binding to Caf1. The minimal region of Caf1 binding site on Ash1 is termed as ‘Caf1 Binding Motif (CBM)’. **C** The sequence conservation of CBM in Ash1s among *Drosophila melanogaster*, *Homo sapiens*, *Mus musculus* and *Danio rerio*. Absolutely conserved residues are highlighted in red, and partially conserved residues in yellow

Ash1 is a multidomain protein containing AWS, SET, post-SET, BRD, PHD and BAH domains [4, 35]. Except for the AT-hook domain, the majority of the conserved domains are concentrated in the C-terminal half of Ash1. AWS, SET and post-SET domains form a catalytic core and the BRD, PHD and BAH domains are located in a proximity to form a histone binding module cluster (Fig. 1A). Although the catalytic core is sufficient to methylate histone H3K36, it exhibits very poor activity, as the substrate binding pocket is blocked by an autoinhibitory loop coming from the post-SET domain [2]. Based on these findings, it was suggested that another element is required to release the autoinhibitory loop of Ash1. Later studies found that Ash1 forms a stable complex with Mrg15 and Caf1 (a.k.a. Nurf55 and p55 for fly and RbAp48/46 in human), termed the AMC complex [11, 26]. Mrg15 is a transcription factor containing Chromo and Mrg domains. The Ash1 and Mrg15 complex shows the same level of the histone H3K36 methyltransferase activity to the AMC complex for recombinant nucleosome substrate in-vitro. Consistent with this, the crystal structures of human ASH1L bound with MRG15 show that the binding of MRG15 destabilizes the autoinhibitory loop and in turn activates ASH1L histone methyltransferase activity [10, 14]. As the Ash1 and Mrg15 complex without Caf1 has been shown to exhibit HMTase activity similar to that of the AMC full complex, the role of Caf1 in the AMC complex remains unclear.

Caf1 was initially identified as p55, the smallest subunit of Chromatin Assembly Factor 1 (CAF1) [30]. Subsequently, Caf1 was identified as a key component of several chromatin modifiers including PRC2 histone methyltransferase, Mi-2/NuRD chromatin remodeler and histone acetyl transferase complexes [5, 9, 13, 19, 22, 33, 38, 41]. Caf1 belongs to the WD40 protein family having a seven-bladed β-propeller structure. Caf1 contains two conserved histone binding sites located on the top and the side of the β-propeller. Caf1 interacts with histone H3, FOG-1 and Aebp2 using the top pocket designated as ‘histone H3 binding pocket’ and interacts with histone H4 helix 1, SUZ12, and MTA1 at the side pocket designated as ‘histone H4 binding pocket’ [1, 12, 15, 27, 31].

It is particularly interesting that Caf1 preferentially binds to the unmodified histone H3 tail and methylation (mono-, di- and tri) of H3K4 significantly decreases its binding to methylated histone H3 to the greatest extent with the trimethylation of H3K4 [27]. Caf1 in PRC2 complex is shown to sense unmodified histone H3 tail to regulate PRC2 activity [27]. Therefore, PRC2 shows significantly lower activity toward H3K4 methylated nucleosomes.

However, the function of Caf1 in the AMC histone H3K36 methyltransferase complex has not been investigated. Therefore, we explored the role of Caf1 in the AMC complex. Specifically, we dissected the interaction between Caf1 and Ash1, showing that Caf1 utilizes the histone H4 binding pocket to interact with Ash1 near the histone reader domain cluster. Furthermore, we showed that Caf1 in AMC also plays a role in sensing unmodified histone H3 to regulate AMC activity in an internucleosomal manner. Our data revealed a delicate mechanism by which the AMC histone H3K36 methyltransferase complex is regulated.

## Results

### Caf1 interacts with the proximal region of the histone binding module cluster in Ash1

Ash1 directly binds to Mrg15 via the N-terminal region of the Ash1 catalytic domain and it also binds to Caf1 in the AMC complex [10, 14]. However, how Caf1 binds to Ash1 has not been investigated. Therefore, we dissected the interaction between Caf1 and Ash1. To identify the Caf1 interaction region on Ash1, we generated a series of truncated constructs of Ash1 and examined their interactions with Caf1 (Fig. 1). First, we divided Ash1 into the N-terminal part (Ash1_N) containing the catalytic domain and the C-terminal part containing the histone binding module cluster (Ash1_C). GST pull-down experiments show that Caf1 binds to Ash1_C. These data reveal that Ash1 uses two distinct regions to interact with Mrg15 and Caf1 (Fig. 1A). To further dissect the Caf1 interaction site in Ash1, we generated four constructs and performed a GST pull-down assay (Fig. 1A). These constructs are an N-terminal region to the histone binding module (Ash1_C1), a BRD (Ash1_C2), a PHD–BAH domain (Ash1_C3) and a C-terminal region (Ash1_C4) to the histone binding module. We found that Caf1 strongly binds to the N-terminal region to the histone binding module cluster (Ash1_C1) and weakly associates with the C-terminus to the reader domains (Ash1_C4). To narrow down the interaction site at the amino acid level, we then continued to trim down the Ash_C1 fragment into smaller fragments (Fig. 1B). Among these fragments, Caf1 binds to the 1582–1616 fragment (Ash1_C1.6). In addition, further trimming down the Ash1_C1.6 fragment substantially abrogated the interaction with Caf1 (Additional file 1: Fig. S1). Therefore, we concluded that Caf1 interacts with the region of 1582–1616 a.a. in Ash1 and we named this minimal binding region as ‘Caf1-binding-motif (CBM)’. We examined the sequence conservation of CBM among species (Fig. 1C). The CBM region is highly conserved among humans, mice, zebrafish and flies, suggesting that Caf1 interacts with Ash1 via CBM in other species as well (Additional file 1: Fig. 2S).

**Fig. 2:**
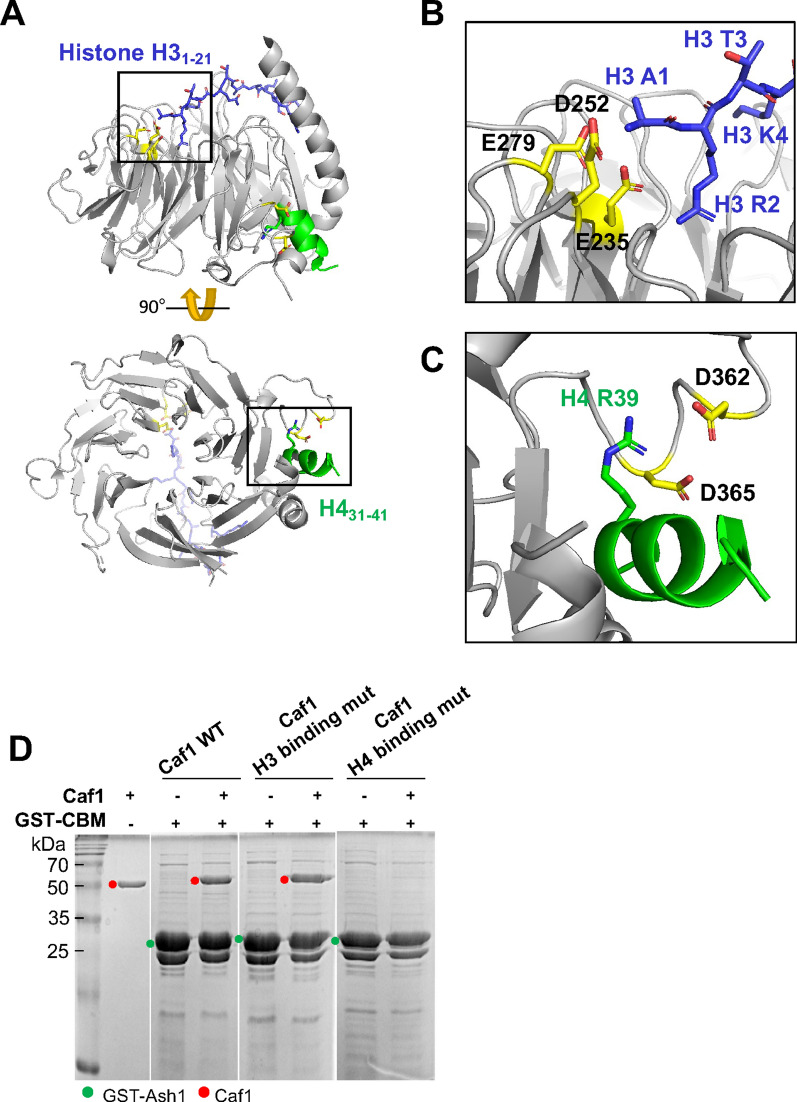
Caf1 utilizes the H4 binding pocket to interact with Ash1_CBM. **A** The crystal structures of Caf1-histone H3_1–21_ complex (blue, PDB ID: 2yba) and Caf1-H4 helix1_3__1–41_ (green, PDB ID: 3c9c) is shown in one Caf1. **B** The histone H3 binding pocket is located at the central pore of Caf1. A1 and R2 residues of H3 N-terminal tail are coordinated with E235, D252 and E279 of Caf1. **C** Caf1 utilizes a pocket located at the side of the WD40 β-barrel structure for H4 binding. The first helix of histone H4_31–41_ is accommodated with the carboxyl groups of D362 and D365, and the backbone carbonyl groups in a loop (366–371 a.a.) in Caf1. **D** GST pulldown assay using GST-CBM and Caf1 and Caf1 mutants. Caf1 H3 binding mut has mutations of E235Q/D252K/E279Q in Caf1, and Caf1 H4 binding mutant has mutations of D362A/D365A in Caf1

### Caf1 utilizes the histone H4 binding pocket for Ash1 interaction

Caf1 contains two histone binding pockets. One pocket recognizes the first helix of histone H4 and is located at the side of WD40 β-propeller structure and the other pocket recognizes histone H3 N-terminal tail and is located at the central pocket (Fig. 2A). It has been demonstrated that the histone H4 binding pocket is very versatile; this pocket also binds to other proteins, including SUZ12 and MTA1, in addition to histone H4 [1, 27]. To examine whether Caf1 utilizes these pockets for Ash1 binding, we generated Caf1s in which the histone binding pockets were mutated. First, we mutated the histone H3 binding surface (E325Q/D252K/E279Q) (Fig. 2B) and examined its binding to the Ash1 CBM fragment (Fig. 2D). These mutations were previously shown to disrupt the interaction between Caf1 and histone H3 without affecting the integrity of Caf1 structure [20]. The mutation in the histone H3 binding pocket of Caf1 did not abrogate the interaction with Ash1. Next, we generated Caf1 mutant where the histone H4 binding pocket was mutated (D362A/D365A) (Fig. 2C). These mutations were also previously shown to disrupt the interaction the first helix of histone H4 and Caf1 without affecting the integrity of Caf1 structure. This Caf1 mutant completely lost the binding to Ash1 (Fig. 2D). These data show that Caf1 utilizes the histone H4 binding pocket to interact with Ash1.

**Fig. 3:**
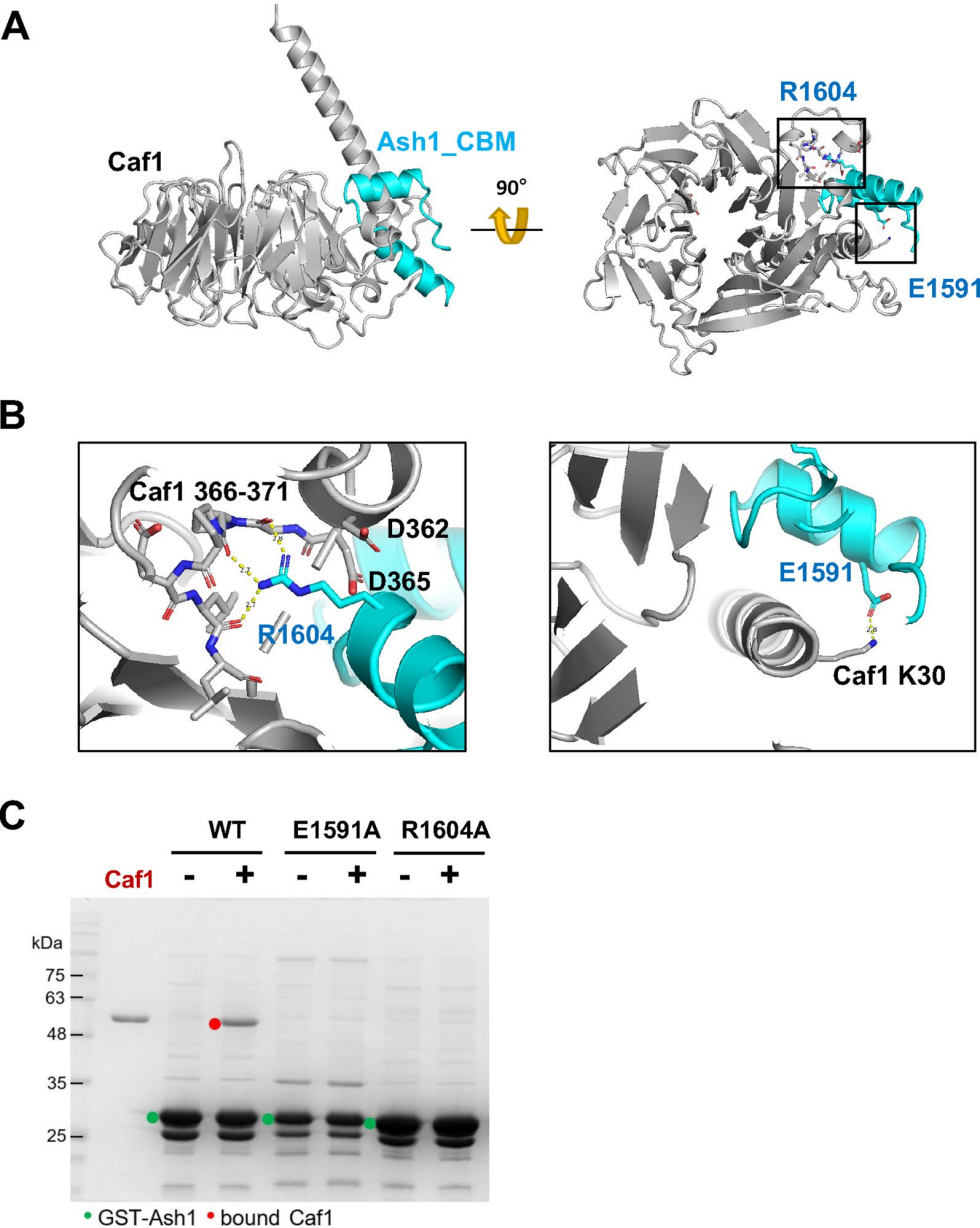
The binding mode of CBM on Caf1. **A** An AlphaFold predicted structural model of Caf1 (grey) bound to CBM (1582–1616 a.a., cyan). The CBM binds to Caf1 histone H4 binding pocket, forming a clip-like structure. **B** Detailed interactions between Caf1 and CBM. R1604 of CBM makes extensive electrostatic interaction with carboxyl groups in the loop (366–371 a.a.) and D362/D365 residues in Caf1 (left panel). In addition, E1591 in CBM makes a salt bridge with K30 in Caf1, further stabilizing the Caf1_CBM interaction. **C** GST pulldown assay using GST-CBM wild-type and mutants for Caf1 binding. GST-CBMs are marked with green dots, and CBM bound Caf1 in red dots

To further examine the binding mode of Caf1 and CBM, we analyzed the Caf1 and CBM interaction using AlphaFold structure prediction. We generated a model of the Caf1-CBM complex by feeding the Caf1 and CBM sequences to the AlphaFold structure prediction platform [18]. Interestingly, the AlphaFold predicted structure shows that CBM forms a helix and binds to the histone H4 binding pocket of Caf1 (Fig. 3A). In addition, the predicted structure of human ASH1L CBM and RbAP48 utilizes a binding mode similar to that of the fly CBM-Caf1 interaction (Additional file 1: Fig. S3). These modelled structures are consistent with our GST-pulldown assay, showing that the mutating this histone H4 binding pocket abrogates the Caf1 and Ash1 interaction (Fig. 2D). In the Caf1-histone H4 complex structure [31], R39 of histone H4 is coordinated by two aspartates (D362 and D365) and the backbone carbonyl groups in the loop (366–371 a.a.). Interestingly, the highly conserved R1604 of Ash1 is coordinated with the same residues including D362 and D365 (Fig. 3B). In addition, other absolutely conserved residues (E1591, V1595, F1601, L1602, N1605 and K1608) among species may be involved in the interaction with Caf1 (Fig. 1C and Additional file 1: Fig. S4). To confirm this interaction mode predicted by the AlphaFold, we generated CBM_E1591A_ or CBM_R1604A_ mutants and examined their binding to Caf1. GST pull-down assays show that these mutants did not bind to Caf1 (Fig. 3C). These data show that Caf1 utilizes the versatile histone H4 binding pocket to interact with Ash1.

### Caf1 senses the unmodified histone H3 tail to regulate AMC HMTase activity

The Caf1 subunit (RbAp48) of PRC2 functions as a sensor for active chromatin marks by recognizing unmodified histone H3. Caf1 preferentially binds to unmodified histone H3 tails and methylated histone H3 (H3K4_me3_) inhibits PRC2-mediated histone H3K27 methylation [27]. Therefore, we sought to determine whether the Caf1 subunit in AMC functions in a similar manner to PRC2. To test this hypothesis, we measured the HMTase activity of AMC with unmodified or H3K4 methylated G5E4 nucleosome arrays. For H3K4 methylated nucleosome, we installed methylated lysine using the methyl-lysine analog (MLA) protocol (Additional file 1: Fig. S5) [29].

**Fig. 4:**
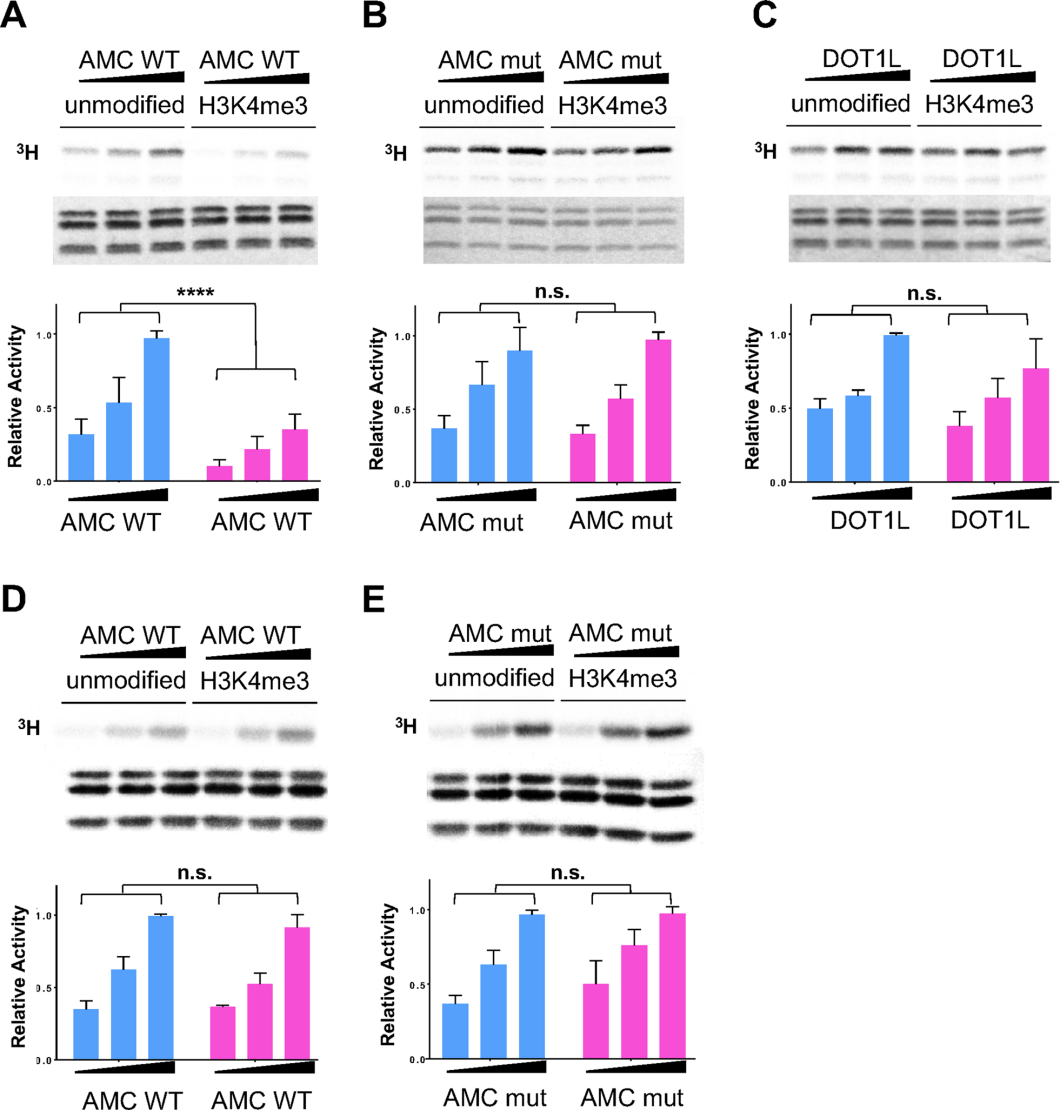
Caf1 in AMC complex senses unmodified histone H3 on nucleosome array. **A**–**C** HMT assay using G5E4 nucleosome array and AMC complex (70, 100 and 140 nM) (top panels). The relative activity of AMC complexes on unmodified nucleosome array is shown in blue, and H3K4me3 nucleosome array in magenta (bottom panels): AMC wild-type (AMC WT), AMC with Caf1 H3 binding mutant (E235Q/D252K/E279Q) (AMC mut) and DOT1L H3K79 methyltransferase (DOT1L) (*n* = 3, ****p value < 0.0001, n.s. = not significant). **D**, **E** HMT assay using mononucleosome with 80 bp linker DNA (top panels) and the relative activity shown in a bar graph (*n* = 3, n.s. = not significant) (bottom panels)

Interestingly, AMC shows significantly lower activity for methylated nucleosomes than for unmodified nucleosomes, suggesting that Caf1 in AMC senses unmodified histone H3 as it does in PRC2 (Fig. 4A). As Caf1 interacts with the unmodified histone H3 tail through the central pore of the WD40 β-barrel structure, we examined whether the H3 binding pocket of Caf1 is utilized to sense unmodified histones in the AMC. We generated mutations (E235Q/D252K/E279Q) in the histone H3 binding surface of Caf1 (Fig. 2B) to abolish the interactions between Caf1 and histone H3. We then measured the AMC HMTase activity. In contrast to wild-type AMC, AMC with the Caf1 H3 binding mutant showed similar activity for both unmodified and methylated nucleosome arrays (Fig. 4B). As both AMC WT and AMC mut show similar activity for both unmodified and modified mononucleosomes, the difference is not due to the difference in the quality of the complexes (Additional file 1: Fig. S6C).

Furthermore, we assessed the quality of the two nucleosome arrays, confirming that this observation is not due to the different quality of the nucleosome arrays using DOT1L histone H3K79 methyltransferase. DOT1L activity was not affected by the presence of methylation in histone H3K4 (Fig. 4C). These data imply that the Caf1 subunit in AMC senses unmodified histone H3K4.

Recently, PRC2 was shown to work on dinucleosomes [23]. Therefore, we examined whether AMC works on dinucleosomes and whether the inhibitory effect of methylated histones on AMC results from internucleosomal phenomena. We generated unmodified and H3K4 methylated mononucleosomes with 147 bp Widom 601 DNA with 80 bp linker DNA at one side and measured the AMC HMTase activity. Both wild-type AMC and the histone H3 binding mutant AMC showed no significant difference for unmodified and methylated mononucleosomes (Fig. 4D and E). As AMC shows similar activity to both unmodified and methylated mononucleosomes, AMC may act on more than one nucleosome; in addition, the inhibitory effect of methylated nucleosomes on AMC activity may result from internucleosomal interactions.

## Discussion

In this study, we dissected the interaction among the subunits of fly Ash1 histone methyltransferase complex, showing that the Caf1 subunit interacts with the highly conserved region named ‘Caf1-Binding-Motif (CBM)’ at the N-terminal proximity to the histone binding module of Ash1 via the Caf1 histone H4 binding pocket. Furthermore, we demonstrated that Caf1 in the Ash1 complex plays a role in sensing unmodified histone H3 to regulate histone methyltransferase activity through internucleosomal interactions.

Ash1 has a histone binding module cluster composed of BRD, PHD and BAH. Interestingly, our data showed that Caf1 binds to CBM located in N-terminal proximity to the histone binding module cluster (Fig. 1). Considering that Caf1 is also a histone binding module, Caf1 may function cooperatively with the BRD–PHD–BAH cluster may function cooperatively to recognize combinatorial histone modifications. CBM is highly conserved among species, suggesting that Ash1s from other species also utilizes the CBM motif for Caf1 interaction (Fig. 1C).

Caf1/p55/Nurf55/RbAp48 is a very versatile protein that functions as a histone binder and as a scaffold to assemble large chromatin modifying complexes and as a histone binder. Our biochemical and structural analysis shows that Caf1 utilizes the histone H4 binding pocket located at the side of the β-propeller structure to interact with the CBM of Ash1. As this pocket is also shown to interact with several other proteins such as SUZ12 and MTA1 (Additional file 1: Fig. S3), it is likely that there are more other proteins interacting with this pocket.

Caf1 also binds to histone H3 tails using the pocket at the top of β-propeller structure. At the histone H3 binding pocket, the ε-amine group of H3K4 is coordinated by two aspartates (E130 and E183). Caf1 exhibit a nanomolar binding affinity (~ 600 nM) toward unmodified histone H3 and a 100-fold lower affinity (> 70 µM for H3Kme3) toward methylated histone H3 tails [27]. This preferential binding ability of Caf1 as a component of PRC2 plays a critical role in sensing unmodified nucleosome substrates. Therefore, PRC2 exhibits substantially lower activity toward H3K4 methylated nucleosome substrates. Interestingly, our data show that AMC histone H3K36 methyltransferase also shows substantially lower activity toward H3K4 methylated nucleosomes than unmodified nucleosomes; in addition, the AMC complex containing Caf1 with the histone H3 binding pocket mutant does not discriminate methylated and unmethylated nucleosomes, implying that Caf1 in the AMC complex functions a similar manner to PRC2.

Both histone H3K36 and H3K4 methylation marks are largely considered as active marks for gene expression. Therefore, it is disintuitive that histone H3K4 methylation inhibits AMC H3K36 methylation activity. However, some H3K4 methylation patterns in the genome are quite distinct and do not overlap with the H3K36 methylation pattern [7]. Therefore, it is plausible that Caf1 in AMC may play a role in discriminating these regions by sensing H3K4 methylation free nucleosomes.

Ash1 possesses a histone binding module cluster that contains BRD, PHD and BAH domains, and Caf1. It is likely that these clusters recognize combinatorial histone modifications. However, the full spectrum of the binding specificity of these domains is not clear. A large-scale structural analysis suggests that the human ASH1L bromo domain interacts with multiple acetylated histones including H3K56ac and H4K59ac [8]. However, the biological role of the interactions has not been investigated. In addition, a recent structural analysis of the ASH1L PHD domain shows that it binds H3K4 methylated peptide with very weak affinity (100–200 µM range) [39] and does not bind to unmodified H3K4 peptide. It is quite interesting that although Caf1 and PHD are in proximity to each other, Caf1 shows strong affinity toward unmodified H3K4 and very weak affinity to methylated H3K4. To add more complexity, the chromodomain of Mrg15, which is a component of AMC, has been shown to bind to methylated histone H3K36 [40]. The binding of Mrg15 to methylated H3K36 may confer Ash1 to propagate H3K36 methylation. The propagation of H3K27methylation by PRC2 through sensing methylated H3K27 was also observed [36]. However, structural studies combined with functional assays will be necessary to precisely understand the contribution of histone modifications to Ash1 activity.

**Fig. 5:**
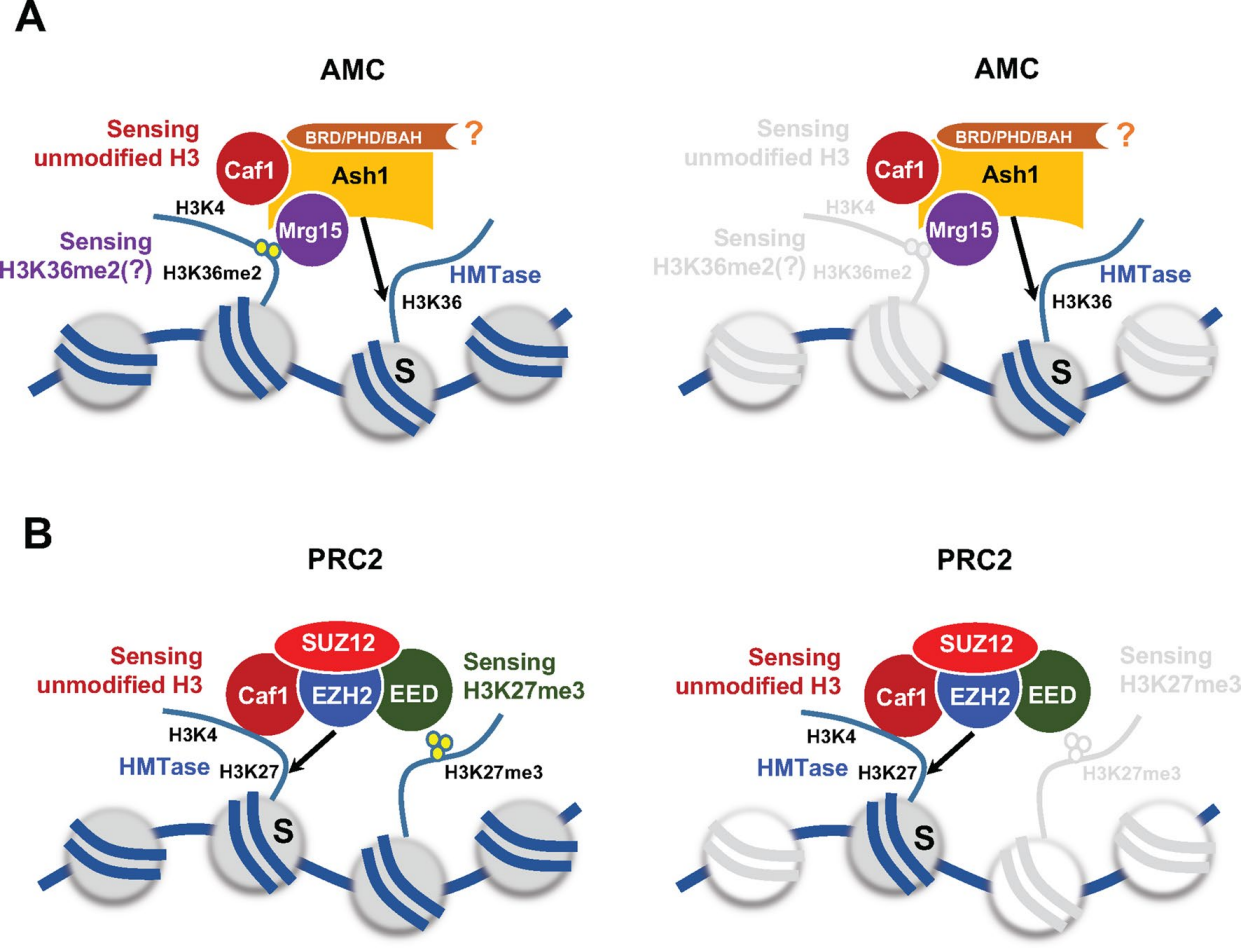
A proposed working model of AMC complex in a comparison with PRC2. **A** In AMC complex, Caf1 senses unmodified histone H3K4, and Mrg15 may interact with di-methylated histone H3K36 in one nucleosome. Ash1 methylates histone H3K36 in the other nucleosome (substrate nucleosome, S). The function of the histone binding module (BRD/PHD/BAH) is not known (indicated with a question mark). **B** In PRC2 complex, Caf1 together with SUZ12 senses unmodified histone H3K4 in one nucleosome, and EED senses methylated histone H3K27 in the other nucleosome. The EZH2 catalytic subunit in PRC2 methylates histone H3K27 in a substrate nucleosome (S)

Chromatin modifying complexes often work on more than one nucleosome. For example, the PRC2 complex is engaged in two nucleosomes [23]. One nucleosome serves as a substrate nucleosome, and the other nucleosome serves as an activating nucleosome; modification of this nucleosome stimulates PRC2 activity. Our data show that the substrate preference of AMC for unmodified nucleosomes was observed only with the use of oligonucleosomes and not mononucleosomes (Fig. 4D). Therefore, the inhibitory effect on AMC activity by the methylated nucleosome likely occurs in an internucleosomal manner. The domain organization in Ash1 is also consistent with the idea that Ash1 engages two nucleosomes. A large flexible region links the catalytic domain and the histone binding module cluster. It is conceivable that the catalytic domain binds to one nucleosome as a substrate and the cluster senses the other nucleosome, which plays a regulatory role. Based on our findings, we propose a working model of AMC complex in a comparison with PRC2 H3K27 methyltransferase complex (Fig. 5).

The activity of Ash1 HMTase seems to be delicately regulated by several factors. First, the catalytic activity is regulated by the autoinhibitory loop. Second, the AMC complex contains a histone binding module cluster, which may recognize specific modifications to regulate the AMC activity. Third, this study reveals that the Caf1 subunit senses the unmodified histone H3 tail to regulate the activity.

Overall, our data imply that the activity of AMC histone H3K36 methyltransferase is highly regulated, and further in vitro and in vivo studies are needed to fully determine how AMC activity is regulated.

## Materials and methods

### Protein expression and purification

Fly Caf1 wild-type, H3 binding pocket mutant (E235Q/D252K/E279Q), and H4 binding pocket mutant (D362A/D365A) mutant were cloned into a modified pFastBac_HTB vector with a 6×-His tag and TEV cleavage site on its N-terminus. It was expressed and purified from Sf9 cells using a baculovirus system. After transfection, cells were harvested in 300 mM NaCl, 50 mM Tris–HCl (8.0), and 5% glycerol buffer and lysed by 4 freeze–thaw cycles. Lysed cells were cleared by 15,000 rpm centrifugation for 2 h. Equilibrated Ni-NTA resin (Qiagen) in the same buffer with cells was subjected to cleared lysates for 2 h and then washed with 5 column volume (CV) of 1 M NaCl and 2 CV of 300 mM NaCl, with 50 mM Tris-HCl(8.0), 20 mM imidazole buffer. Resin-bound Caf1 was eluted by 100 mM imidazole elution buffer. The 6× His-tag on Caf1 was cut by TEV protease, and then Caf1 purification was performed with HiTrap Q HP (Cytiva) and Superdex 200 26 600 (Cytiva) in buffer containing 150 mM NaCl and 50 mM Tris–HCl (8.0). To purify the AMC complex, 6× His-tagged Ash1 (1041–2226 a.a.), full-length Mrg15, and full-length Caf1 were coinfected into Sf9 cells using baculovirus, and the cells were harvested in 300 mM, NaCl, 50 mM Tris-HCl (8.0), and 5% Glycerol buffer. Cells were lysed by 4 freeze–thaw cycles and cleared by 15,000 rpm centrifugation for 2 h. Equilibrated Ni-NTA resin (Qiagen) was incubated in cleared lysate for 2 h and washed with 300 mM–1 M (3CV)–300 mM NaCl buffer with 50 mM Tris-HCl (8.0) 20 mM imidazole buffer. Bound proteins were eluted with 300 mM NaCl, 50 mM Tris-HCl (8.0), 5% glycerol and 100 mM imidazole buffer, and the affinity tags were cut by TEV protease. The cut His-tag and TEV protease were removed by recapturing with Ni-NTA beads. Purification of AMC protein was then performed by HiTrap Q HP (Cytiva) and Superdex 200 26 600 (Cytiva) in the buffer containing 300 mM NaCl and 50 mM Tris-HCl (8.0).

### Nucleosome reconstitution

Recombinant *Xenopus laevis* histone and 601 positioning DNA sequences with an 80 bp extension on one side (+ 80 DNA) were expressed and purified from *Escherichia coli* BL21 (DE3) pLysS and DH5α, respectively, and the remaining purification procedures were followed by a published protocol [17]. For the reconstitution of histone octamer, four types of histones were dissolved in unfolding buffer and mixed with H2A:H2B:H3:H4 = 1.1:1.1:1:1 ratio in molar concentration. The buffer was changed to 2 M NaCl solution through dialysis overnight at 4 °C, and the histone H2A/H2B dimer was discarded through a Superdex 200 size exclusion column (Cytiva). After reconstitution of the histone octamer, purified 601 DNA and octamer were mixed in a 1.0 to 1.6 ratio depending on the types of DNA or octamer. The salt concentration in the mixture dropped slowly from 2 M NaCl to 250 mM NaCl using a dual peristaltic pump, and finally 0 mM NaCl by dialysis.

### GST pull-down assay

For the GST pull-down assay, Ash1 fragments were cloned into the pGEX-4T-1 vector. Cloned GST-tagged Ash1 fragments were expressed in the *E. coli* BL21 (DE3) RILP strain at 18℃ in the presence of 0.5 mM IPTG for 18 h, and harvested in 150 mM NaCl and 50 mM Tris-HCl (8.0) buffer. Cells were lysed by sonication and cleared by centrifugation at 13,300 rpm for 10 min. Equilibrated GST resin (Qiagen) was introduced to the cleared lysate and incubated for 2 h to bind with GST-bound Ash1 fragments, and then washed several times with 150 mM NaCl 50 mM Tris-HCl (8.0) buffer. After washing, 200 µL of 0.1 mg/mL purified Caf1 was added to GST resin and incubated for 1 h, and the unbound Caf1 were washed out. Resin-bound proteins were analyzed by SDS-PAGE.

### Methyltransferase assay (HMTase assay)

For the HMTase assay, 1 µM of wild-type AMC and mutant AMC were mixed with 100 mM NaCl, 50 mM Tris-HCl (8.0) and 150 ng/µL of G5E4 nucleosome array or 1 µM of + 80 mononucleosome. Tritium SAM (PerkinElmer) was mixed with 10× HMTase buffer (500 mM Tris-HCl (9.0), 50 mM MgCl_2_, 40 mM DTT) and added to the reaction mixture. The reaction was performed at 25 °C for 40 min, stopped by heating to 65 °C for 5 min, and then SDS buffer was added and boiled in 95 °C for 5 min. Proteins were separated by 16% SDS PAGE and transferred to PVDF membranes by semi-dry transfer. The membrane was exposed to the imaging plate overnight, and the tritium signals were detected by FUJI-BAS. The intensities of the detected bands were measured by the ImageLab program.

## Supplementary Information

**Additional file 1:** **Figure S1.** Caf1 Binding Motifis a minimal binding site of Caf1. A. A series of the truncation constructs of CBM. Strong binding is indicated with ‘++’, weak binding with ‘+’ and no binding with ‘−’ based on GST-pulldown experiments. B. GST pulldown assay using GST-CBM fragments and Caf1. GST-tagged CBMs are marked in green and Caf1 marked with red dots. Weakly binding Caf1s are marked with red empty dots. **Figure S2.** Sequence alignment of Ash1. The fly Ash1 1227–2226 sequence conservation among Drosophila melanogaster, Homo sapiens, Mus musculus and Danio rerio. Absolutely conserved residues are highlighted in red and partially conserved residues in yellow. **Figure S3.** Alpha Fold predicted model of Caf1 and Ash1_CBM. A. Alpha Fold predicted model of *Drosophila melanogaster* Caf1 and Ash1_CBM sequence. Caf1 colored in grey, and Ash1_CBM colored in cyan. B. Alpha Fold predicted model of *Homo sapiens* RbAp48 and ASH1L_CBM sequence. RbAp48 colored in grey, and ASH1L_CBM colored in dark cyan. **Figure S4.** Conserved residues on Ash1_CBM extensively coordinate with Caf1 H4 binding pocket. Molecular interaction between L1602, N1605, and K1608 on Ash1. L1602 is found in close proximity to hydrophobic residues on Caf1 I373 and F372. N1605 and K1608 create salt bridges with the Caf1 L371 backbone carboxyl group, and the D415 side chain carbonyl group, respectively. In addition, V1595 and F1601 on Ash1 interact with Caf1 residues. V1591, F1601 and Caf1 I27, I35 side chains positioned in close proximity to stabilize the Ash1-Caf1 binding via hydrophobic interaction. **Figure S5.** Western blot of MLA crosslinked H3K4me mimic nucleosome. Western blot data of unmodified and H3K4me3 MLA nucleosomes using α-H3K4me3 antibody and α-H3 antibody. **Figure S6.** Triplicate autoradiograms of AMC WT, AMC mutant and DOT1L HMTase assays. A, Triplicate HMT assays using unmodified/H3K4me3 G5E4 nucleosome array with WT and H3 binding mutant AMC complexwith a Commassie stained gel of histones. B, Triplicate HMT assay using unmodified/H3K4me3 G5E4 nucleosome array with WT AMC and DOT1L histone methyltrasferase with a Commassie stained gel of histones. C, Triplicate HMT assay using unmodified/H3K4me3 mono nucleosome with WT and the H3 binding mutant AMC complex with a Commassie stained gel of histones.** Figure S7.** Proteins, which bind to the Caf1 H4 pocket, has conserved Arg. A. Superimposition of crystal structures of Caf1 perimeter binding proteins: H4, MTA1, Suz12and the Alpha Fold modelled CBM. D362 and D365 residues in Caf1 and the loopcoordinate with the conserved Arg. B. The orientation of conserved Args in the Caf1 binding proteins.

## Data Availability

All materials will be available upon request to J.S. (songj@kaist.ac.kr).
