# Supplementary material for: Caf1 regulates the histone methyltransferase activity of Ash1 by sensing unmodified histone H3
Source: Epigenetics Chromatin. 2023 Apr 29;16:15. doi: 10.1186/s13072-023-00487-6 (PMC10148413; doi:10.1186/s13072-023-00487-6)
Supplement: Supplementary file 1 — Additional file 1: Figure S1. Caf1 Binding Motifis a minimal binding site of Caf1. A. A series of the truncation constructs of CBM. Strong binding is indicated with ‘++’, weak binding with ‘+’ and no binding with ‘−’ based on GST-pulldown experiments. B. GST pulldown assay using GST-CBM fragments and Caf1. GST-tagged CBMs are marked in green and Caf1 marked with red dots. Weakly binding Caf1s are marked with red empty dots. Figure S2. Sequence alignment of Ash1. The fly Ash1 1227–2226 sequence conservation among Drosophila melanogaster, Homo sapiens, Mus musculus and Danio rerio. Absolutely conserved residues are highlighted in red and partially conserved residues in yellow. Figure S3. Alpha Fold predicted model of Caf1 and Ash1_CBM. A. Alpha Fold predicted model of Drosophila melanogaster Caf1 and Ash1_CBM sequence. Caf1 colored in grey, and Ash1_CBM colored in cyan. B. Alpha Fold predicted model of Homo sapiens RbAp48 and ASH1L_CBM sequence. RbAp48 colored in grey, and ASH1L_CBM colored in dark cyan. Figure S4. Conserved residues on Ash1_CBM extensively coordinate with Caf1 H4 binding pocket. Molecular interaction between L1602, N1605, and K1608 on Ash1. L1602 is found in close proximity to hydrophobic residues on Caf1 I373 and F372. N1605 and K1608 create salt bridges with the Caf1 L371 backbone carboxyl group, and the D415 side chain carbonyl group, respectively. In addition, V1595 and F1601 on Ash1 interact with Caf1 residues. V1591, F1601 and Caf1 I27, I35 side chains positioned in close proximity to stabilize the Ash1-Caf1 binding via hydrophobic interaction. Figure S5. Western blot of MLA crosslinked H3K4me mimic nucleosome. Western blot data of unmodified and H3K4me3 MLA nucleosomes using α-H3K4me3 antibody and α-H3 antibody. Figure S6. Triplicate autoradiograms of AMC WT, AMC mutant and DOT1L HMTase assays. A, Triplicate HMT assays using unmodified/H3K4me3 G5E4 nucleosome array with WT and H3 binding mutant AMC complexwith a Commassie stained gel of histon [file 13072_2023_487_MOESM1_ESM.pdf]

Figure S1

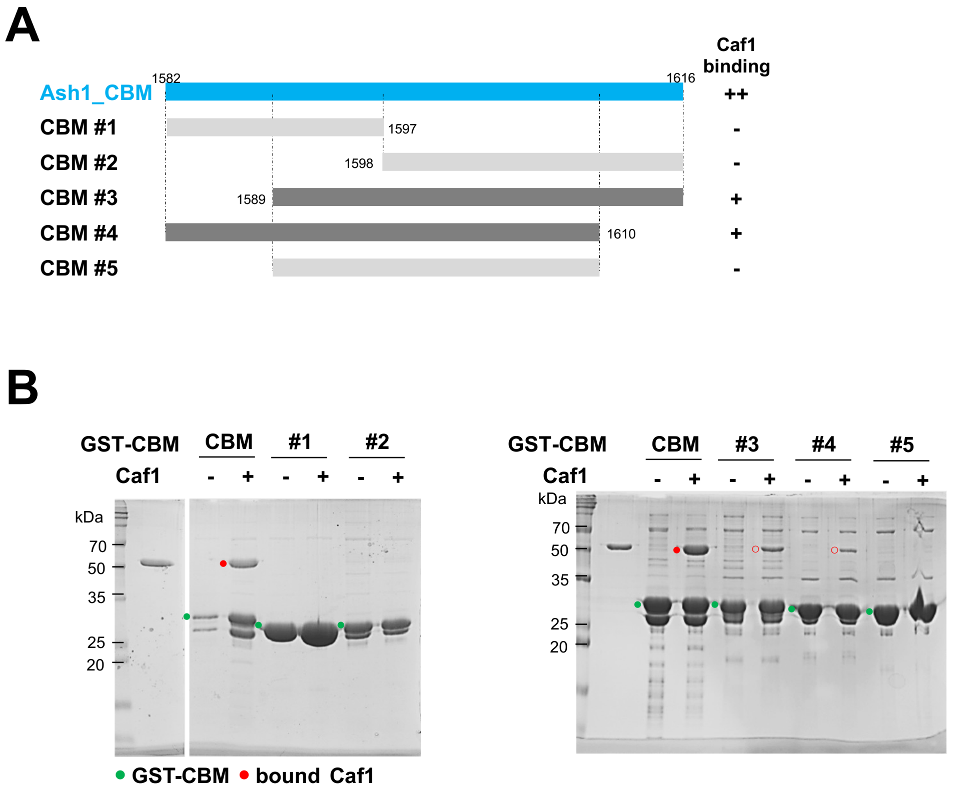

Figure S1. Caf1 Binding Motif (CBM) is a minimal binding site of Caf1.
A. A series of the truncation constructs of CBM. Strong binding is indicated with '++', weak binding with '+' and no binding with '-' based on GST-pulldown experiments. **B.** GST pulldown assay using GST-CBM fragments and Caf1. GST-tagged CBMs are marked in green and Caf1 marked with red dots. Weakly binding Caf1s are marked with red empty dots.

Figure S2

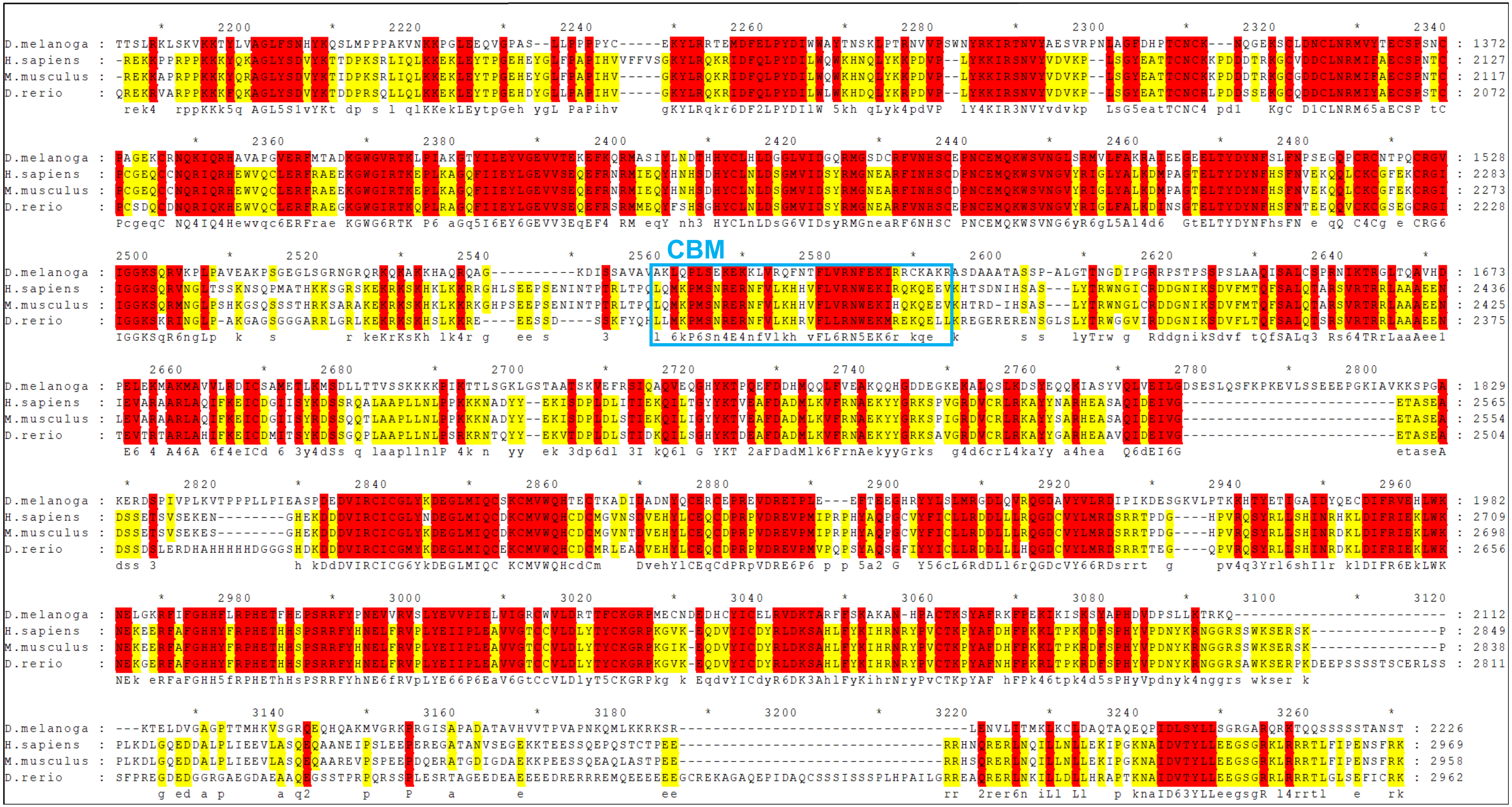

Figure S2. Sequence alignment of Ash1.
The fly Ash1 1227-2226 sequence conservation among *Drosophila melanogaster, Homo sapiens, Mus musculus* and *Danio rerio*. Absolutely conserved residues are highlighted in red and partially conserved residues in yellow.

Figure S3

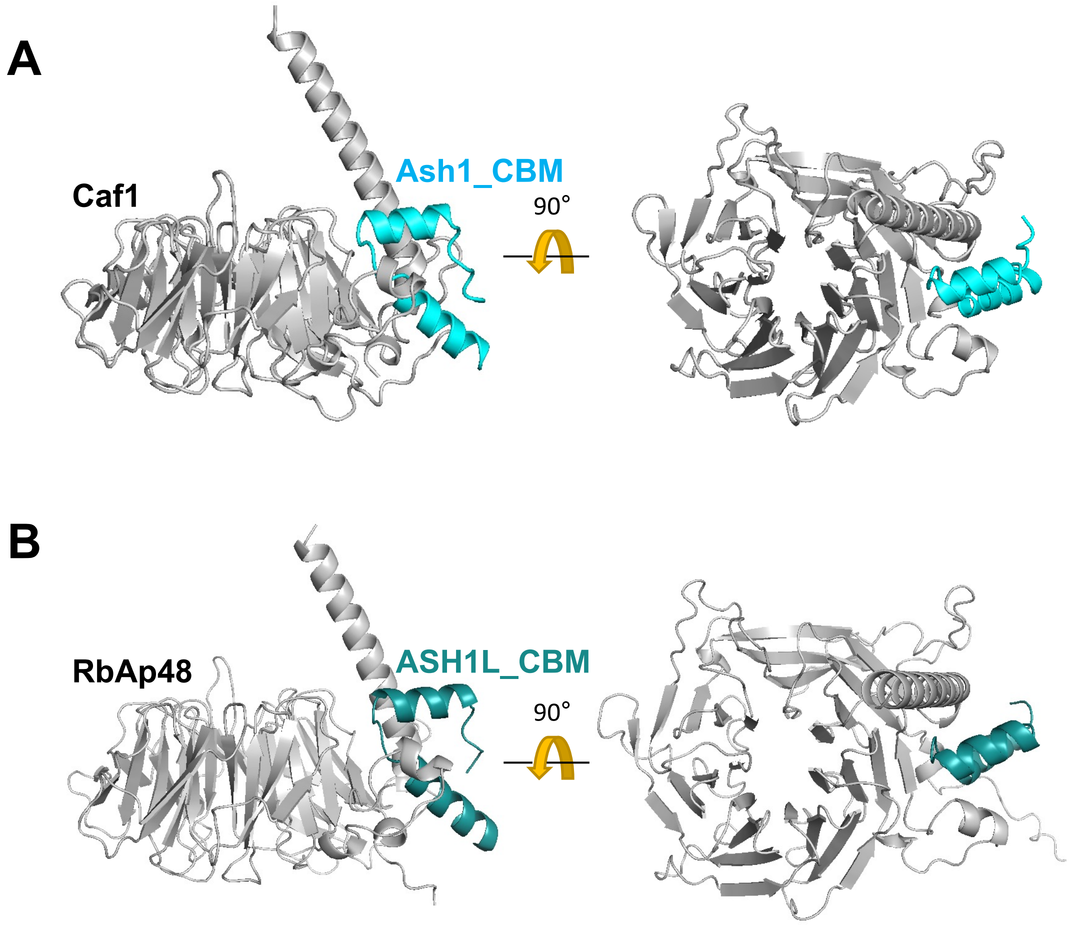

Figure S3. Alphafold predicted model of Caf1 and Ash1_CBM.
A. Alphafold predicted model of Drosophila melanogaster Caf1 and Ash1_CBM sequence. Caf1 colored in grey, and Ash1_CBM colored in cyan. **B.** Alphafold predicted model of Homo sapiens RbAp48 and ASH1L_CBM sequence. RbAp48 colored in grey, and ASH1L_CBM colored in dark cyan.

Figure S4

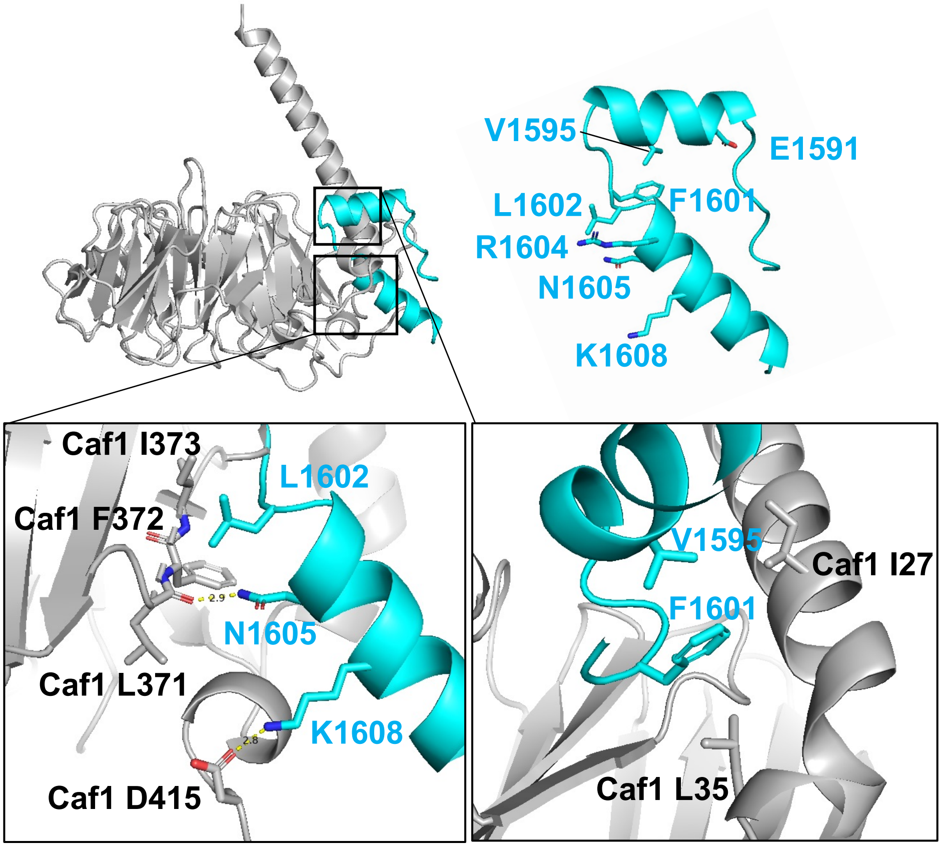

Figure S4. Conserved residues on Ash1_CBM extensively coordinate with Caf1 H4 binding pocket.
Molecular interaction between L1602, N1605, and K1608 on Ash1. L1602 is found in close proximity to hydrophobic residues on Caf1 I373 and F372. N1605 and K1608 create salt bridges with the Caf1 L371 backbone carboxyl group, and the D415 side chain carbonyl group, respectively. In addition, V1595 and F1601 on Ash1 interact with Caf1 residues. V1591, F1601 and Caf1 I27, I35 side chains positioned in close proximity to stablize the Ash1-Caf1 binding via hydrophobic interaction.

Figure S5

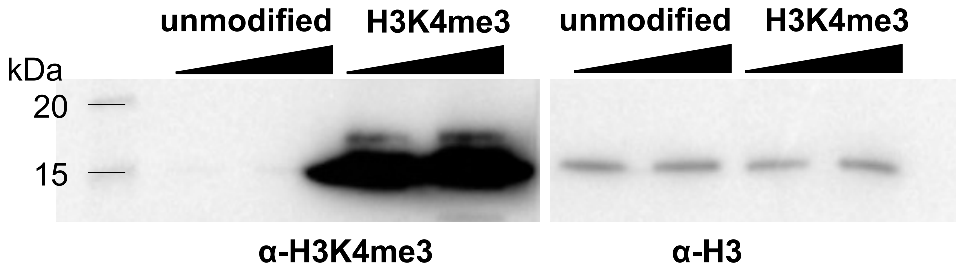

Figure S5. A western blot of MLA crosslinked H3K4me mimic nucleosome
Western blot data of unmodified and H3K4me3 MLA nucleosomes using α-H3K4me3 antibody and α-H3 antibody.

Figure S6

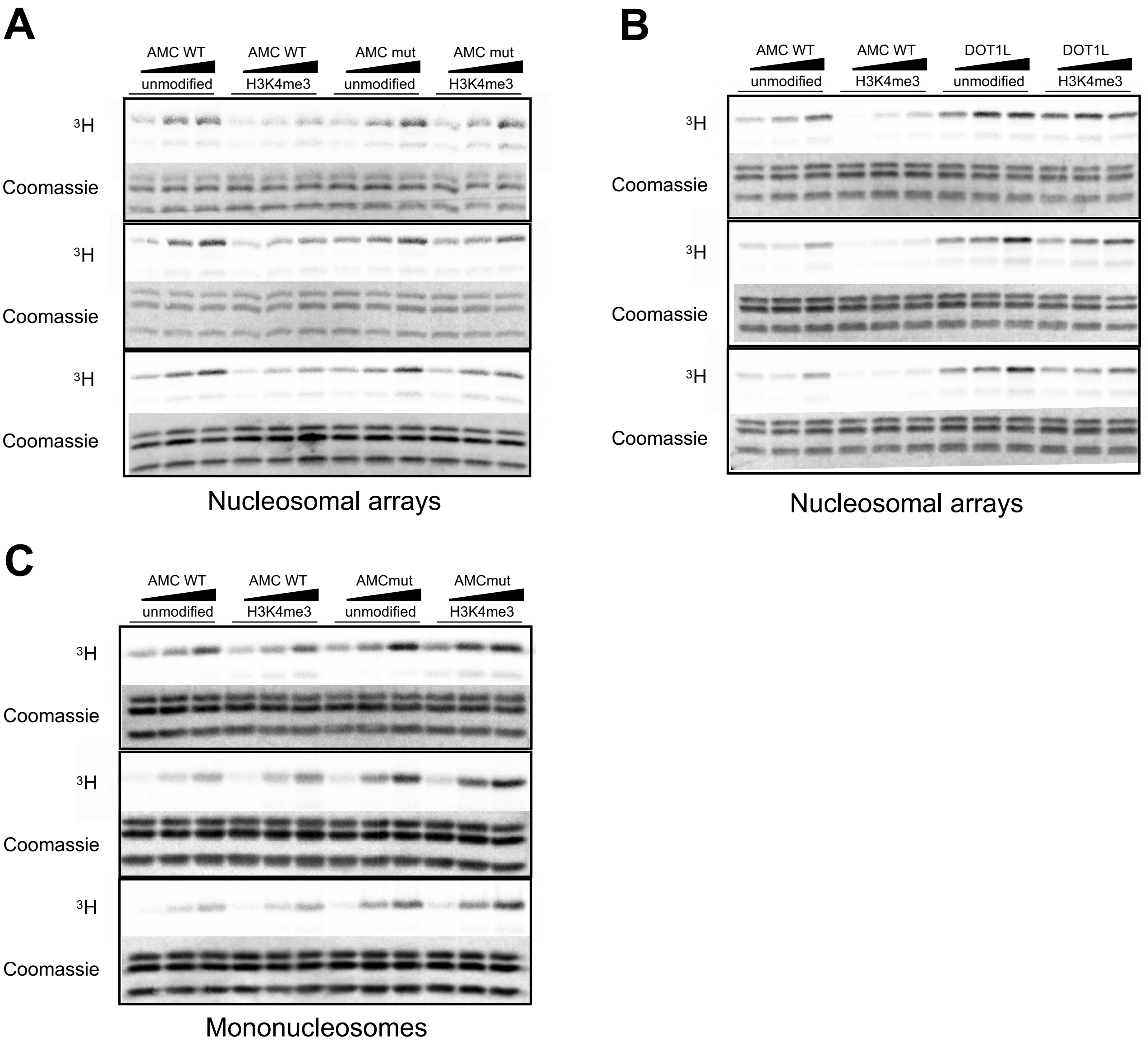

Figure S6. Triplicate autoradiograms of AMC WT, AMC mutant and DOT1L HMTase assays.

A, Triplicate HMT assays using unmodified/H3K4me3 G5E4 nucleosome array with WT and H3 binding mutant AMC complex (70, 100 and 140 nM) with a Commassie stained gel of histones. **B**, Triplicate HMT assay using unmodified/H3K4me3 G5E4 nucleosome array with WT AMC and DOT1L histone methyltrasferase with a Commassie stained gel of histones. **C**, Triplicate HMT assay using unmodified/H3K4me3 mono nucleosome with WT and the H3 binding mutant AMC complex with a Commassie stained gel of histones.

Figure S7

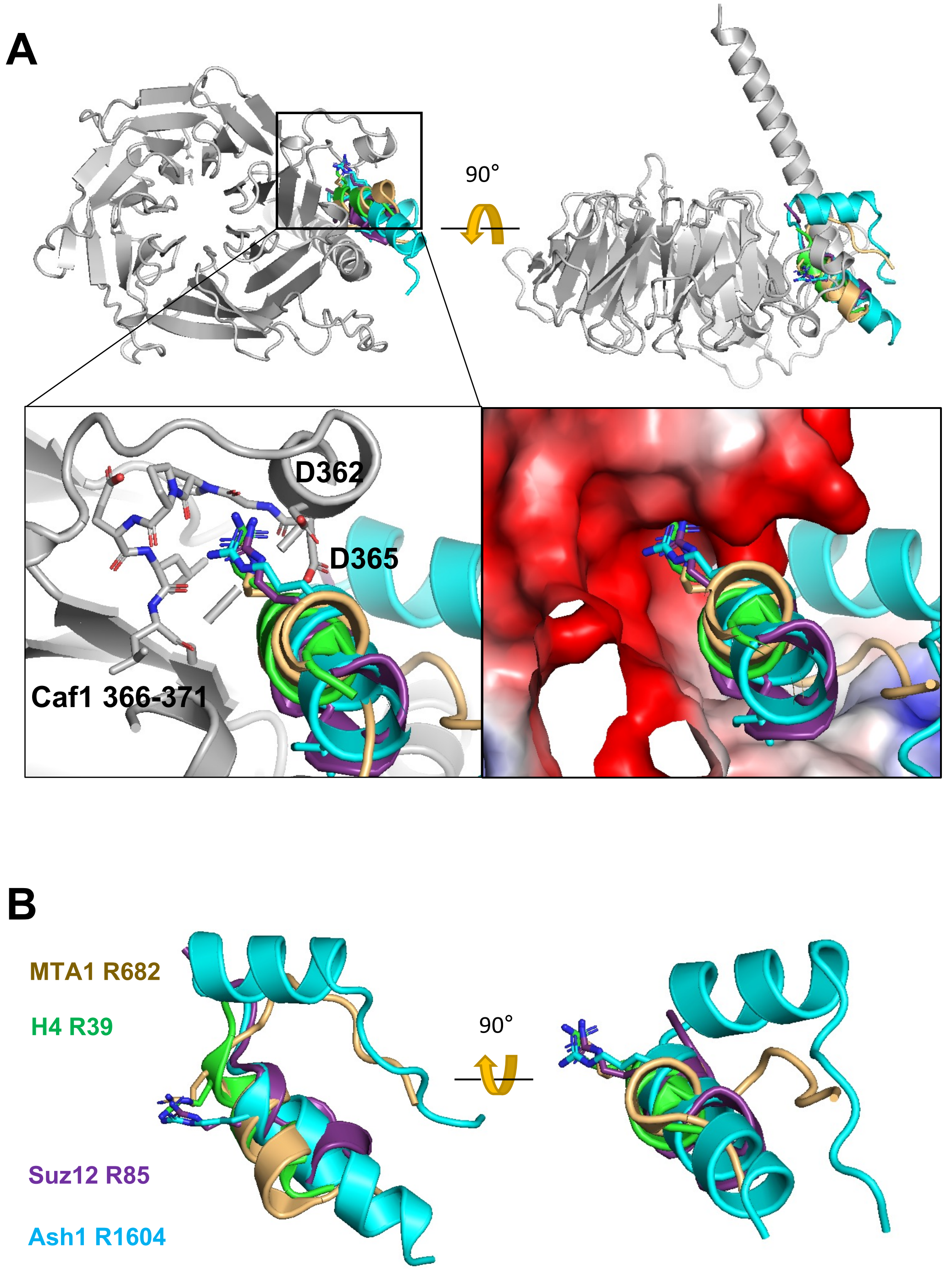

Figure S7. Proteins, which bind to the Caf1 H4 pocket, have conserved Arg.
A. Superimposition of crystal structures of Caf1 perimeter binding proteins : H4 (green, PDB ID : 3c9c), MTA1 (yellow, PDB ID : 4pc0), Suz12 (purple, PDB ID : 2yb8) and the alphafold modelled CBM (cyan). D362 and D365 residues in Caf1 and the loop (366-371 a.a.) coordinate with the conserved Arg. **B.** The orientation of conserved Args in the Caf1 binding proteins.
